# Post-colonoscopy appendicitis: a case report

**DOI:** 10.1093/jscr/rjab285

**Published:** 2021-07-14

**Authors:** Kostas Tepelenis, Christos K Stefanou, Stefanos K Stefanou, Periklis Tsoumanis, Konstantina M Ntalapa, Vasiliki Galani, George Gogos-Pappas, Konstantinos Vlachos

**Affiliations:** Department of Surgery, University Hospital of Ioannina, Ioannina, Greece; Department of Surgery, General Hospital of Ioannina, “G. Xatzikosta”, Ioannina, Greece; Department of Surgery, General Hospital of Ioannina, “G. Xatzikosta”, Ioannina, Greece; Department of Ophthalmology, University Hospital of Ioannina, Ioannina, Greece; Department of Nursing, University of Ioannina, Ioannina, Greece; Department of Anatomy-Histology-Embryology, Medical School, University of Ioannina, Ioannina, Greece; Department of Surgery, University Hospital of Ioannina, Ioannina, Greece; Department of Surgery, University Hospital of Ioannina, Ioannina, Greece

## Abstract

Appendicitis after colonoscopy is rare, with an estimated incidence of 3.8 cases per 10 000 colonoscopies. Herein, we report a 56-year-old female who visited the emergency department with a history of diffuse abdominal pain and nausea 8 h after a screening colonoscopy. Abdominal examination disclosed deep tenderness at Mc Burney point and positive Rovsign’s sign. Laboratory studies revealed elevated white blood cells and neutrophils (WBC 15.37 K/Ul and NEUT 86.5%) with normal C-reactive protein (5 mg/l). The initial diagnosis was acute appendicitis, which was confirmed by the ultrasonographic findings. The patient was admitted to the surgical department, and a laparoscopic appendectomy was performed. Post-colonoscopy appendicitis is increasingly recognized as a complication after colonoscopy in the last decade. Early recognition is vital in preventing morbidity and mortality. It may also be worthwhile to include appendicitis after colonoscopy as a possible complication during the consent before the procedure.

## INTRODUCTION

Colonoscopy is usually performed to diagnose or correct a problem in the large or distal small intestine. Its clinical applications have significantly increased in recent years [1]. Intestinal perforation and bleeding are the most typical complications of colonoscopy, but their incidence is exceptionally low [1, 2].

Post-colonoscopy appendicitis is extremely infrequent, with a reported incidence of 3.8 cases per 10 000 colonoscopies [3, 4]. Nowadays, it has become more recognizable, and endoscopists need to be alert that appendicitis is a possible complication of colonoscopy. According to our knowledge, 53 case reports have been published until now. Here, we describe the case of a 56-year-old patient who developed appendicitis after colonoscopy.

## CASE REPORT

A 56-year-old female visited the emergency department with a history of diffuse abdominal pain and nausea 8 h after a screening colonoscopy. There was no history of diarrhea, constipation or vomiting. The patient was afebrile and reported no significant past medical or surgical history. In the morning, the patient got a screening colonoscopy. The preparation was excellent, requiring no cecal washing. The colonoscope was advanced to the cecum without great difficulty. It was not impacted against the appendiceal orifice. No biopsy or polypectomy was performed.

**Figure 1:**
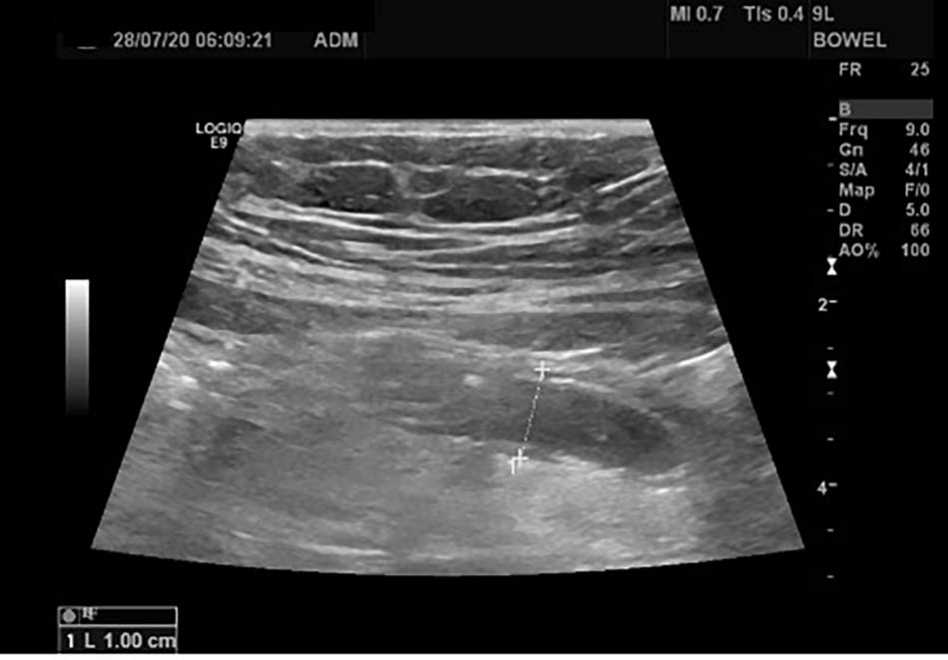
Abdominal ultrasound: The appendix is enlarged (diameter 1 cm) and non-compressible. Inflammation of the adjacent fat is also observed.

Abdominal examination disclosed deep tenderness at Mc Burney point and positive Rovsign’s sign, whereas rebound tenderness in the right iliac fossa, psoas sign and obturator sign were all negative. Laboratory studies revealed elevated white blood cells and neutrophils (white blood cell count 15.37 K/Ul and neutrophils 86.5%) with normal C—reactive protein (5 mg/l). The initial diagnosis was acute appendicitis confirmed by the ultrasonographic findings: enlarged noncompressible blind tubular structure in the right iliac fossa (diameter 1 cm) periappendiceal fat stranding. No free fluid was found (Fig. 1).

The patient was admitted to the surgical department, and a laparoscopic appendectomy was performed. The appendix was identified, exposed and noticed to be enlarged, hardened but without inflammation of the cecum and distal ileum. There was no free fluid, mesenteric lymphadenopathy or Meckel’s diverticulum. Laparoscopic appendectomy was carried out using a harmonic scalpel (Ethicon Endo-Surgery, Johnson & Johnson, Cincinnati, USA). No antibiotics were administered post-operatively. The patient recovered uneventfully, and she was discharged after 2 days. The histological report evinced the diagnosis of acute appendicitis: a heavy focal infiltration of the mucosa by neutrophils that spread out of the appendix wall, especially in the serosa, focal edema, and congested vessels of medium and largesize.

## DISCUSSION

Colonoscopy is a widely used endoscopic procedure for diagnosing, surveillance or screening various colorectal diseases. Despite its rare complications, colonoscopy is regarded as a safe technique. Perforation is the most severe complication, with a reported incidence of 0.016–0.8% for diagnostic colonoscopy and up to 5% for therapeutic colonoscopy. Bleeding is another significant complication happening in 2.4 cases per 1000 colonoscopies. Other rare complications encompass post-polypectomy syndrome, splenic injury, small bowel perforation, appendicitis, cholecystitis, pancreatitis, cecal volvulus and mesenteric ischemia [1, 2].

Post-colonoscopy appendicitis is considered an extremely rare complication after colonoscopy. It was first described by Houghton and Aston in 1988 and is being cited with increasing frequency recently [5]. The incidence has been calculated from systematic reviews and ranges from 3.8 cases per 10 000 colonoscopies [3, 4, 6]. The mean age of onset is 55 years (range 24–84), and there is a male predominance. Colonoscopy is usually performed for screening, while most patients have a colonoscopy with an additional procedure such as polypectomy or endoscopic mucosal resection (EMR). The time of diagnosis ranges from few hours to 10 days. The vast majority of patients is diagnosed within the first 48 h post-colonoscopy [7, 8].

The exact mechanism by which colonoscopy induces appendicitis remains unclear. Several theories have been proposed, including pre-existing subclinical appendicitis, the introduction of a fecalith into the appendix, barotrauma resulting from increased airway pressure, direct trauma or exposure of the mucosa to the residual glutaraldehyde type solution for cleaning the endoscope [6, 8–11].

The clinical manifestation of post-colonoscopy appendicitis is the same as common acute appendicitis. Patients usually experience pain in the right lower quadrant or diffuse abdominal pain with or without related symptoms like nausea, vomiting and fever. On physical examination, findings of fever, tachycardia and peritonism (rebound tenderness, guarding) should raise suspicion of abdominal sepsis. The main concern in all patients who experience pain after colonoscopy is perforation of the colon, primarily when polypectomy is carried out. Unfortunately, complications such as bowel perforation and post-polypectomy syndrome may mimic post-colonoscopy appendicitis [7, 8].

Computed tomography is the imaging modality of choice for the diagnosis of post-colonoscopy appendicitis, as post-colonoscopy pain can be brought about by retained gas, colonic spasm or complications [12]. Ultrasound might also distinguish between post-colonoscopy appendicitis and other complications after colonoscopy, whereas plain abdominal films display non-specific signs, which are usually attributed to bowel perforation and post-colonoscopy syndrome, causing a delay in the diagnosis and management of post-colonoscopy appendicitis [8].

The management of post-colonoscopy appendicitis is not different from common acute appendicitis. In recent years, laparoscopy has gained much popularity and has become the preferred approach for uncomplicated and complicated appendicitis [13]. Advantages of laparoscopic appendectomy encompass the shorter length of hospital stay, earlier return to normal activities, less postoperative pain, an earlier start of oral intake, and lower wound infections [14, 15]. Another benefit of laparoscopy is the rapid scrutiny for evidence of bowel perforation, and if present, it can be easily converted to an exploratory laparotomy [7]. In lacking equipment or expertise surgeons, open appendectomy remains a safe and quick alternative [8].

## AUTHORS’ CONTRIBUTIONS

K.T. studied conception and design, drafting of manuscript; P.T. studied conception and design, drafting of manuscript; S.K.S. did literature search and acquisition of data; C.K.S. did literature search and acquisition of data; K.M.N. performed analysis and interpretation of data; V.G. performed analysis and interpretation of data; G.G-P. did critical revision and K.V. did final approval of the version to be submitted.

All the authors agreed to be accountable for all aspects of the work in ensuring that questions related to the accuracy or integrity of any part of the work are appropriately investigated and resolved.
